# Impact of hepatic impairment and renal failure on the pharmacokinetics of linezolid and its metabolites: contribution of hepatic metabolism and renal excretion

**DOI:** 10.1128/aac.01892-24

**Published:** 2025-04-14

**Authors:** Jinyao Liu, Yingying Pang, Wenyan Li, Juanjuan Sun, Yujie He, Yonghong Guo, Jing Dong

**Affiliations:** 1 Postgraduate Training Base at Shanghai Pudong New Area Gongli Hospital, Ningxia Medical University, Shanghai, China; 2 Department of Pharmacy, Shanghai Pudong New Area Gongli Hospital 639328 , Shanghai, China; 3 Department of Pathology, Shanghai Pudong New Area Gongli Hospital 639328 , Shanghai, China; 4 Department of Infectious Diseases, Shanghai Pudong New Area Gongli Hospital 639328 , Shanghai, China; Bill & Melinda Gates Medical Research Institute, Cambridge, Massachusetts, USA

**Keywords:** linezolid, pharmacokinetics, hepatic impairment, renal failure, hepatic metabolism, drug transporter

## Abstract

Linezolid, an oxazolidinone antibiotic, is used in patients with liver or kidney disease. However, the effects and mechanisms of hepatic impairment or renal failure on the pharmacokinetics of linezolid and its metabolites (PNU-142586 and PNU-142300) remain unclear. We used carbon tetrachloride-induced impaired hepatic function and 5/6 nephrectomy-induced renal failure rat models to investigate linezolid and metabolite pharmacokinetics. Isolated primary rat hepatocytes were used to evaluate the impact of hepatic impairment or renal failure on linezolid metabolism. Uptake and efflux transport studies were also conducted. The influence of hepatic impairment or renal failure on the pharmacokinetics of linezolid and two metabolites did not differ between intragastric gavage and intravenous administration in rats. Linezolid did not accumulate in the brain, heart, lung, liver, kidney, and small intestinal tissues of the hepatic impairment or renal failure rats. And PNU-142300 did not accumulate in the liver or kidney tissue. Compared to the isolated normal rat hepatocytes, the *in vitro* hepatic clearance of linezolid in hepatic impairment and renal failure rat hepatocytes decreased by 61.3% and 44.1%, respectively. Organic anion transporting polypeptide (OATP)1B1, OATP1B3, OATP2B1, Na+-taurocholate co-transporting polypeptide (NTCP), organic anion transporter (OAT)1, OAT3, multidrug resistance-associated protein 2 (MRP2), or multidrug resistance protein 1 (MDR) did not mediate linezolid transport. Hepatic impairment primarily increases linezolid exposure through reduced hepatic metabolism, whereas renal failure increases both linezolid and two metabolites exposure through reduced hepatic metabolism and renal glomerular filtration. These findings guide adjusting the dose of linezolid in patients with hepatic and renal insufficiency.

## INTRODUCTION

Liver disease and kidney disease are complex, progressive diseases, and a global health concern (1, 2). Patients with liver or kidney disease have impaired humoral and cellular immune responses that increase their risk of infections (3, 4). Infections are a major source of high morbidity and mortality, and antibiotics are frequently prescribed for these patients (5, 6).

**Fig 1:**
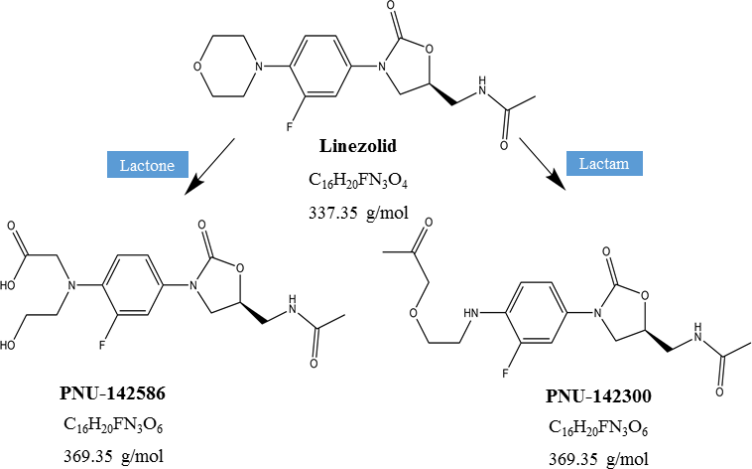
The main metabolic process of linezolid.

Linezolid is an oxazolidinone antibiotic with good activities against vancomycin-resistant *Enterococci*, methicillin-resistant *Staphylococcus aureus*, and multidrug-resistant *Mycobacterium tuberculosis* (7–9). Linezolid exhibits good tissue penetration and can be used for the treatment of severe infections such as pneumonia, intra-abdominal infections, and skin infections (7, 10). It is generally well tolerated and its predominant adverse drug reaction is myelosuppression, including reversible anemia and thrombocytopenia (11). Linezolid is rapidly absorbed after oral administration, and its bioavailability is nearly 100%. Maximum concentration (C_max_) is usually achieved between 0.5 and 2 h. The plasma protein binding rate is 31%, and the apparent volume of distribution (V_d_) is 40–50 L (12, 13). The total clearance (CL) is 80 mL/min and it is excreted by both renal and non-renal routes, with an elimination half-life (t_1/2_) of 5–7 h. Linezolid is primarily metabolized through non-enzymatic oxidation of the morpholine ring into two main inactive ring-opened carboxylic acid metabolites, namely aminoethoxyacetic acid metabolite (PNU-142300) and hydroxyethyl glycine metabolite (PNU-142586) (Fig. 1) (12, 14, 15). Approximately 50% of the administered linezolid dose is excreted in the urine as these two metabolites, and 35% is excreted as the parent drug. The formation of PNU-142586 is the rate-limiting step in the clearance of linezolid in humans, whereas the formation of PNU-142300 is the rate-limiting step for the clearance of linezolid in rats (12).

Organic anion transporters (OATs), organic cation transporters (OCTs), organic anion transporting polypeptides (OATPs), Na^+^-taurocholate co-transporting polypeptide (NTCP), multidrug resistance proteins (MDRs), multidrug resistance-associated proteins (MRPs), breast cancer resistance protein (BCRP), and multidrug and toxic compound extrusion proteins (MATEs), distributed in the enterocytes, hepatocytes, and renal proximal tubular cells, are involved in the disposition and elimination of many drugs (16–18). Linezolid is not a substrate for OCT1, OCT2, OCT3, MATE1, and MATE2-K (19, 20), while it is an inhibitor of OAT1, OAT3, OATP1B1, OATP1B3, and OATP2B1. In addition, linezolid is not an inhibitor of OCT1, OCT2, OCT3, MAT1, and MATE2-K (20–22). However, the specific transporters involved in the intestinal absorption, hepatic uptake, and renal excretion of linezolid remain unclear.

Hepatocyte inflammatory response, matrix deposition, parenchymal cell death, and angiogenesis result in liver disease. Hepatic impairment reduces metabolic enzyme and transporter activities, inhibits metabolite formation and elimination, and alters drug pharmacokinetics. Carbon tetrachloride (CCl_4_) has been widely used as a chemical inducer of experimental hepatic impairment (23). Tubular cell stress, tubular cell loss, interstitial inflammation, tubular atrophy, and ischemia lead to renal failure (24). Renal failure may delay the elimination of parent drugs and their metabolites (25). The 5/6 nephrectomized (5/6 Nx) rat model has been widely used as a surgically induced renal failure model (26). Linezolid is used in patients with liver or kidney disease. The standard clinical dose of linezolid for oral or intravenous (i.v.) administration is 600 mg twice daily in healthy adults. Although data are limited, it is generally accepted that mild to moderate liver disease or any degree of renal impairment does not significantly affect linezolid pharmacokinetics, and no dose adjustments are required for these patients (27–29). However, liver or kidney injury has been recently reported to be associated with higher linezolid exposure and thrombocytopenia (30–33). Effects of impaired hepatic function or renal failure on the pharmacokinetics of linezolid, PNU-142586, and PNU-142300 have not yet been fully elucidated, and mechanisms were unclear. There is no theoretical basis to support dose adjustment of linezolid in patients with hepatic impairment or renal failure.

The aims of this study were as follows: (i) to clarify the effects of impaired hepatic function or renal failure on the pharmacokinetics of linezolid, PNU-142586, and PNU-142300 using a CCl_4_-induced hepatic-impaired rat model and a 5/6 Nx treatment renal failure rat model following intragastric gavage (i.g.) or i.v. administration of linezolid; (ii) to assess the impact of impaired hepatic function and renal failure on the metabolism of linezolid using isolated primary rat hepatocytes; and (iii) to identify the drug transporters that are involved in the disposition of linezolid. Our findings provide insights into optimizing linezolid dosing regimens in patients with hepatic impairment or renal failure.

## MATERIALS AND METHODS

### Materials

Linezolid (99.8%) and chloramphenicol (internal standard, IS) were purchased from Aladdin Biochemical Technology Co., Ltd. (Shanghai, China). PNU-142300 was purchased from Shen Zhen H&D Pharmaceutical Technology Co., LTD (Shenzhen, China). PNU-142586 was purchased from Toronto Research Chemicals (Toronto, Canada). CCl_4_, urethane, and dimethyl sulfoxide were purchased from MedChem Express (Monmouth Junction, NJ, USA). In addition, 7-hydroxycoumarin, testosterone, P-aminohippuric acid (PAH), estrone-3-sulfate (ES), N-methyl quinidine (NMQ), cyclosporine A (CsA), estradiol-17β-D-glucuronide (E17G), probenecid, sodium taurocholate (TCA), rifampicin, benzbromarone, ethylene glycol tetraacetic acid (EGTA), and fetal bovine serum (FBS) were purchased from Sigma-Aldrich (St. Louis, MO, USA). Dulbecco’s modified Eagle’s medium (DMEM) was purchased from Gibco (Billings, MT, USA). Phosphate-buffered saline (PBS) and Hanks’ balanced salt solution (HBSS) were purchased from Invitrogen Life Technologies (Carlsbad, CA, USA). The BCA protein assay kit was obtained from Thermo Fisher (Waltham, MA, USA). Acetonitrile, methanol, and acetic acid were all high-performance liquid chromatography (HPLC) grade and purchased from Sigma-Aldrich (St. Louis, MO, USA). All the other chemicals and solvents were of analytical grade.

### Animals

Male Sprague-Dawley rats (8–9 weeks) weighing 180–230 g were purchased from SPF Biotechnology Co., Ltd. (Beijing, China) (Animal license: SCXK (Jing) 2019-0010) and housed under a 12 h light/dark cycle. The animals were provided with water and a chow diet. Prior to the pharmacokinetic experiments, the rats were fasted for 12 h with free access to water. The protocols for animal experiments were approved by the Institutional Animal Care and Use Committee of Shanghai Pudong New Area Gongli Hospital. The experiments were conducted in accordance with the National Institutes of Health (NIH) Guide for the Care and Use of Laboratory Animals (NIH Publication No. 8023, revised 1978).

### Cell lines and membrane vesicles

Human embryonic kidney 293 (HEK293) cells stably transfected with human OATP1B1, OATP1B3, OATP2B1, NTCP, OAT1, OAT3, empty vector (mock cells), and membrane vesicles isolated from *Spodoptera frugiperda*-derived Sf9 cells containing human MDR1 or MRP2 were commercially obtained from GenoMembrane Co., Ltd. (Japan) and used under a license agreement. OATP1B1-, OATP1B3-, OATP2B1-, NTCP-, OAT1-, and OAT3-transfected HEK293 cells were cultured in DMEM supplemented with 10% FBS, 100 U/mL penicillin-streptomycin at 37°C with 95% relative humidity atmosphere containing 5% CO_2_. All cells were seeded in 24-well plates at a density of 4 × 10^5^ cells/well and cultured for 1–2 days until they reached 80%–90% confluence. Prior to the initiation of uptake studies, the cells were washed twice with HBSS buffer at 37°C. Membrane vesicles were stored at −80°C and thawed at 37°C prior to the initiation of the studies.

### Induction of hepatic impairment or renal failure rat models

Hepatic impaired rat models were induced by intraperitoneal (i.p.) injection of CCl_4_ (dissolved in 50% soybean oil solution) at a dose of 2.5 mL/kg for 24 h before linezolid administration (34). The control group rats were intraperitoneally injected with equal volumes of soybean oil. Renal failure was induced in rats by subjecting them to the 5/6 Nx procedure 2 weeks before linezolid administration. The upper and lower poles of the left kidney were removed under urethane anesthesia (1 g/kg; administered by i.p. injection). One week later, the right kidney was completely excised. Rats in the control group underwent a sham operation (35). Serum creatinine (CREA), blood urea nitrogen (BUN), aspartate aminotransferase (AST), alanine aminotransferase (ALT), alkaline phosphatase (ALP), lactate dehydrogenase (LDH), and total bilirubin (TBIL) levels were measured using commercial kits (Shanghai Enzyme-linked Biotechnology Co., Ltd., Shanghai, China) according to the protocols described by the manufacturer before pharmacokinetic experiments.

After the hepatic impairment rats and renal failure rats were euthanized, the liver and the remaining left kidney were collected and fixed in 10% neutral buffered formalin. The tissues were then embedded in paraffin and cut into 3 µm thick sections, stained with hematoxylin and eosin (H&E) for histopathological evaluation. Morphological changes in the H&E sections were observed using a semi-motorized fluorescence microscope (BX53, Olympus Co., Ltd., Tokyo, Japan).

### Pharmacokinetic experiments

The rats were first divided into two groups: i.v. or i.g. administration groups. These two groups were further divided into the following four subgroups (*n* = 5): soybean oil-treated control rats, CCl_4_-induced rats, sham-operated control rats, and 5/6 Nx rats. Linezolid was dissolved in 5% dimethyl sulfoxide containing 0.9% normal saline for i.v. or i.g. administration. The rats were intragastrically administered 63 mg/kg linezolid or intravenously injected 25 mg/kg linezolid via the tail vein. Blood samples (200 µL) were collected from the retro-orbital venous plexus into heparinized tubes at 0 (pre-dose), 0.25, 0.5, 0.75, 1, 2, 3, 4, 6, 8, 12, and 24 h after i.g. administration, and at 0 (pre-dose), 0.083, 0.25, 0.5, 0.75, 1, 2, 3, 4, 6, 8, 12, and 24 h after i.v. administration. Plasma samples were separated by centrifugation at 6,000 × *g* for 5 min at 4°C and stored at −80°C until further analysis using HPLC. Rats were housed in metabolic cages, and urine samples were collected at 0 (pre-dose), 0–1, 1–2, 2–4, 4–6, 6–8, 8–12, and 12–24 h after linezolid administration and stored at −80°C. Linezolid, PNU-142586, and PNU-142300 were analyzed using HPLC.

### Tissue distribution experiments

The rats were first divided into two groups: i.v. or i.g. administration groups. These two groups were further divided into the following three subgroups (*n* = 4): control rats, CCl_4_-induced rats, and 5/6 Nx rats. Linezolid was dissolved in 5% dimethyl sulfoxide containing 0.9% normal saline. Blood samples (200 µL) were collected from the retro-orbital venous plexus at 0.5, 1, and 4 h after i.g. (63 mg/kg) or i.v. (25 mg/kg) administration of linezolid. The rats were euthanized immediately after blood sampling. The brain, heart, lungs, liver, kidneys, and small intestine were collected. The collected tissues were washed and homogenized in normal saline (three-fold volume of each sample weight) using a multi-sample tissue grinder (Tissuelyser-48L; Shanghai Jingxin Industrial Development Co. Ltd., Shanghai, China). After centrifugation of the homogenate samples at 12,000 × *g* for 10 min at 4°C, the supernatants were stored at −80°C. Linezolid, PNU-142586, and PNU-142300 were analyzed using HPLC.

### Primary rat hepatocyte isolation and incubation with linezolid

Primary rat hepatocytes were isolated from normal, CCl_4_-induced, and 5/6 Nx rats according to the previously described two-step collagenase method (36). Rats were anesthetized with urethane (1 g/kg), their abdomens were opened, and their portal veins were cannulated with a needle. The liver was perfused with HBSS solution (without calcium and magnesium ions) containing 5 mM EGTA at a rate of 50–60 mL/min using a peristaltic pump until the blood in the liver was removed. HBSS solution containing 7.5 mg/50 mL collagenase was perfused for 20 min until the liver was completely softened and the surface collapsed. The liver was gently removed and placed in a precooled HBSS solution containing 10% FBS. Hepatocytes were eluted and centrifuged at 500 × *g* for 5 min, and the supernatant was removed to obtain primary rat hepatocytes. Isolated primary rat hepatocytes were washed three times and suspended in DMEM containing 10% FBS, 100 µg/mL streptomycin, 100 U/ml penicillin, and 10 µg/mL insulin at pH 7.65. At this stage of preparation, hepatocyte viability of greater than 82% was demonstrated using trypan blue exclusion.

Freshly isolated rat hepatocytes at a density of 1.0 × 10^6^ cells/well were placed in plates pre-coated with collagen and incubated for 18–24 h in a humidified incubator at 37°C and 5% CO_2_. The rat hepatocytes were then incubated with CYP450 probe substrate testosterone (1 µM), UGT probe substrate 7-hydroxycoumarin (1 µM), or linezolid (1 µM) in fresh medium, respectively. Control incubations were performed in the absence of hepatocytes to test the substrate stability. Three replicate samples (1 mL each) were collected after 1, 2, 4, and 6 h of incubation. Then, 800 µL of methanol was immediately added, and the samples were transferred to a freezer (−20°C) to terminate metabolism. Hepatocytes were quickly washed three times with ice-cold HBSS and then lysed by the addition of 300 µL of purified water. Linezolid, PNU-142586, and PNU-142300 were analyzed using HPLC-MS/MS.

### Uptake transport studies of linezolid

All uptake tests were performed in triplicates. The activities of transporter-transfected HEK293 cells were evaluated using relevant positive substrates and inhibitors. E17G (5 µM, OATP1B1 and OATP1B3), ES (2 µM, OATP2B1), ES (5 µM, OAT3), TCA (10 µM, NTCP), and PAH (20 µM, OAT1) were used as substrates of the transporters. The inhibitors for each transporter were rifampicin (200 µM, OATP1B1 and OATP1B3), erlotinib (10 µM, OATP2B1), CsA (20 µM, NTCP), and probenecid (200 µM, OAT1 and OAT3).

The uptake study was carried out by adding 0.2 mL of HBSS containing linezolid (5 µM) with or without a specific inhibitor to the cells and incubating for 10 min at 37°C. The cells were washed three times with ice-cold HBSS buffer. Then, 0.3 mL of distilled water was added to each well, and the cells were frozen and thawed with liquid nitrogen (−196°C) three times to completely lyse the cells. The lysate was precipitated with methanol and centrifuged at 12,000 × *g* for 5 min, and the intracellular concentration of linezolid was determined using HPLC-MS/MS. The total protein concentration was measured using a BCA Protein Assay Kit. The uptake rates of HEK293-OATP1B1, HEK293-OATP1B3, HEK293-OATP2B1, HEK293-NTCP, HEK293-OAT1, HEK293-OAT3, and mock cells were calculated. We considered that active transport occurred when the uptake ratio of the transporter was ≥2, and the uptake ratio was ≤0.5 in the presence of an inhibitor at a concentration of at least 10 times that of inhibition constants.

### Efflux transport studies of linezolid

All efflux tests were performed in triplicates. The activities of membrane vesicles were evaluated using relevant positive substrates and inhibitors. NMQ (5 µM, MDR1) and E17G (5 µM, MRP2) were used as transporter substrates. The inhibitors for each transporter were CsA (200 µM, MDR1) and benzbromarone (200 µM, MRP2).

A total of 10 µL of membrane vesicles (0.05 mg protein) was pre-incubated with linezolid (5 µM) with or without specific inhibitors. The assay was initiated by adding 5 µL (10 mM) of ATP or AMP reagent to the mixture, followed by incubation at 37°C for 5 min. Transport was then terminated later by washing the vesicles with 0.2 mL of ice-cold HBSS five times, followed by lysis with 50 µL of 80% methanol and centrifugation at 12,000 × *g* for 2 min. The vesicle concentration of linezolid was quantified using HPLC-MS/MS.

### Quantitative analysis of linezolid, PNU-142586, and PNU-142300

Plasma, urine, and tissue samples of linezolid, PNU-142586, and PNU-142300 were analyzed using a Waters E2695 HPLC system (Waters, Milford, MA, USA). Chromatographic separation was performed using the SinoPak BEH T-C_18_ column (4.6 mm × 150 mm, 5 µm, Hi-tech Zone, China) at 35°C. A photodiode array detector was set at a wavelength of 254 nm. The mobile phase consisted of 0.1% (vol/vol) formic acid in water (A) and acetonitrile (B). The gradient conditions were as follows: 0–4.0 min, 14%–86% B; 4.0–5.0 min, 80% B; 5.0–5.7 min, return to 14% B; 5.7–8.0 min, equilibrium. The flow rate was 1.0 mL/min.

Plasma and tissue homogenate samples (100 µL) were mixed with 10 µL of IS (50.0 mg/L) and 200 µL of acetonitrile, followed by vortex-mixing for 3 min. The mixture was centrifuged at 15,000 × *g* for 15 min at 4°C. Then, 5 µL of the supernatant was analyzed. The urine samples were diluted and directly injected for measurement. The analysis method of linezolid, PNU-142586, and PNU-142300 showed a good linear response over the concentration range of 0.25–50.0 mg/L (r^2^ > 0.999) in plasma and tissue homogenate samples. In addition, it showed a good linear response over the concentration range of 1.0–100 mg/L (r^2^ > 0.999) in urine samples. The intra- and inter-assay precision and inaccuracy were all <10%.

An AB Sciex 4000 Mass Spectrometer (AB Sciex, Framingham, MA, USA) and Shimadzu LC 20 series (Shimadzu, Kyoto, Japan) were used for HPLC-MS/MS. Multiple reaction monitoring was used for drug quantification in positive ion mode (m/z 338.0→m/z 294.0 for linezolid, m/z 370.2→m/z 324.2 for PNU-142586 and m/z 370.2→m/z 328.0 for PNU-142300). The Phenomenex Synergi Hydro-RP column (30 mm × 2 mm, 4 µm, Phenomenex Inc., Torrance, CA, USA) was used for chromatographic separation. The mobile phase consisted of 0.1% (vol/vol) formic acid in water (A) and 0.1% (vol/vol) formic acid in acetonitrile (B). The gradient conditions were as follows: 0–1.4 min, 15%–80% B; 1.4–1.8 min, 80% B; 1.8–1.81 min, return to 15% B; 1.81–2.1 min, equilibrium. The flow rate was 450 µL/min. Data acquisition and analysis were performed using the Analyst software (version 1.4.1, AB Sciex).

### Data analysis

The area under the plasma concentration-time curve from 0 to infinity (AUC_0-∞_), time to maximum concentration (t_max_), C_max_, t_1/2_, CL, and V_d_ were calculated by non-compartmental model analysis using the Phoenix 8.1.0 WinNonlin software (Pharsight Corp, Princeton, NJ, USA). The renal clearance (CL_r_), *in vitro* hepatic clearance (CL_int, *in vitro*_), and bioavailability (F) were calculated using the following equations:

CL_r_ = $\frac{A_{\text{total}}}{{\text{AUC}}_{0-\infty }}$

CL_int, in vitro_ (mL/min/kg) = $\frac{0.693}{t_{1/2}}$×$\frac{1}{\text{Hepatocyte concentration}}$×scale factor

F (%) = $\frac{D_{iv}\times {\text{AUC}}_{0-\infty ,\;ig}}{D_{ig}\times {\text{AUC}}_{0-\infty ,\;iv}}$

*A*_total_ is the total cumulative amount excreted in urine over 24 h. *D* is the administered dose. The scale factor is 4,680 (10^6^ cells/kg). All data are expressed as mean ± standard deviation (SD). Statistical analysis was performed using Student’s two-tailed unpaired *t*-test or nonparametric Mann-Whitney U test with SPSS Statistics 19.0 (SPSS Inc., Chicago, IL, USA). Statistical significance was set at *P* < 0.05.

## RESULTS

### Biochemical parameters and histopathological examination of rats

Biochemical parameters of the soybean oil-treated control, CCl_4_-induced, sham-operated control, and 5/6 Nx rats are shown in Table 1. The levels of serum TBIL, ALT, AST, and LDH in CCl_4_-induced rats were 8.40-, 6.92-, 10.7-, and 1.86-fold higher, respectively, than those in the soybean oil-treated control rats. There was no significant difference in serum BUN and CRE levels between the soybean oil-treated control and CCl_4_-induced rats. The levels of serum BUN and CRE in 5/6 Nx rats were 4.15- and 5.36-fold higher, respectively, than those in sham-operated control rats, whereas TBIL, AST, ALT, and LDH levels did not differ. Alterations in biochemical parameters after CCl_4_-induced and 5/6 Nx treatment were consistent with those reported previously (35, 37).

**TABLE 1:** Biochemical parameters of control, CCl_4_-induced, and 5/6 Nx rats^*a*^

Biochemical parameters	Soybean oil-treated control rats	CCl_4_-induced rats	Sham-operated control rats	5/6 Nx rats
TBTL (μmol/L)	0.5 ± 0.2	4.2 ± 2.0^**^	0.6 ± 0.2	0.6 ± 0.3
ALT (U/L)	60.5 ± 27.4	418.8 ± 129.4^**^	67.7 ± 32.9	58.1 ± 12.3
AST (U/L)	225.1 ± 97.4	2399.5 ± 203.2^**^	229.4 ± 96.9	214.9 ± 110.6
ALP (U/L)	247.4 ± 91.3	257.1 ± 94.3	288 ± 86.1	244.9 ± 74.7
LDH (U/L)	1333.7 ± 191.6	2477.6 ± 220.8^**^	1252.3 ± 202.0	1054.4 ± 169.8
BUN (μmol/L)	4.9 ± 1.0	6.1 ± 1.5	4.6 ± 1.2	19.1 ± 4.3^**^
CREA (μmol/L)	15.7 ± 7.8	17.1 ± 7.3	16.0 ± 4.1	85.8 ± 16.2^**^

^*a*^ Data are expressed as mean ± SD (*n* = 28 for each group). TBIL, total bilirubin; ALT, alanine aminotransferase; AST, aspartate aminotransferase; ALP, alkaline phosphatase; LDH, lactate dehydrogenase; CREA, serum creatinine; BUN, blood urea nitrogen. ^**^P<0.01 compared with the control group.

**Fig 2:**
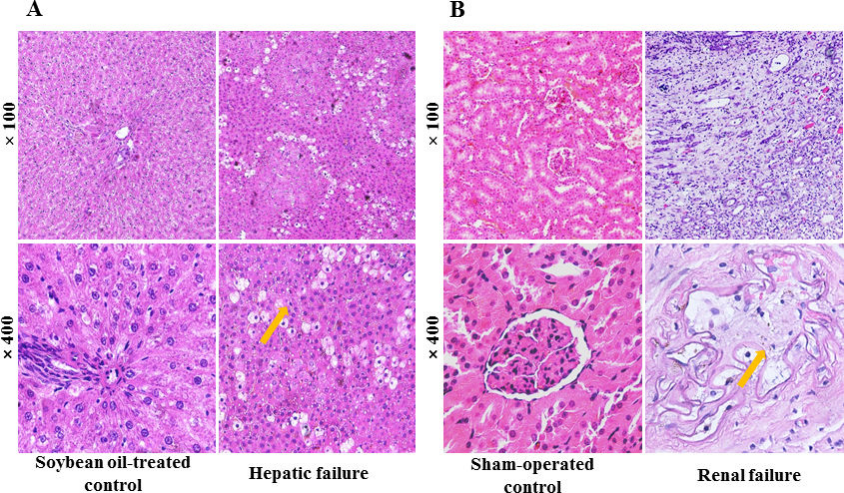
Histopathological evaluation of liver and kidney tissue using H&E staining. Liver tissue stained with HE (**A**) and kidney tissue stained with HE (**B**).

The histopathological results of H&E staining of the liver and kidney tissues are shown in Fig. 2. CCl_4_-induced rats showed hepatocyte degeneration and necrosis, formation of fibrous tissue infiltrated with inflammatory cells, and distortion of the central venules. 5/6 Nx Rats exhibited a blurred glomerular structure, glomerular atrophy and necrosis, disorganized tubular arrangement, dilated lumen, epithelial cell degeneration and necrosis, cytoplasmic lysis, or swelling of the epithelial cell nucleus. The cortical area around the peripheral membrane was heavily infiltrated by fibrous tissue proliferation and inflammatory cells. Therefore, our results demonstrated that hepatic impairment and renal failure rat models were successfully established.

### Effects of impaired hepatic function or renal failure on the pharmacokinetics of linezolid, PNU-142586, and PNU-142300

**Fig 3:**
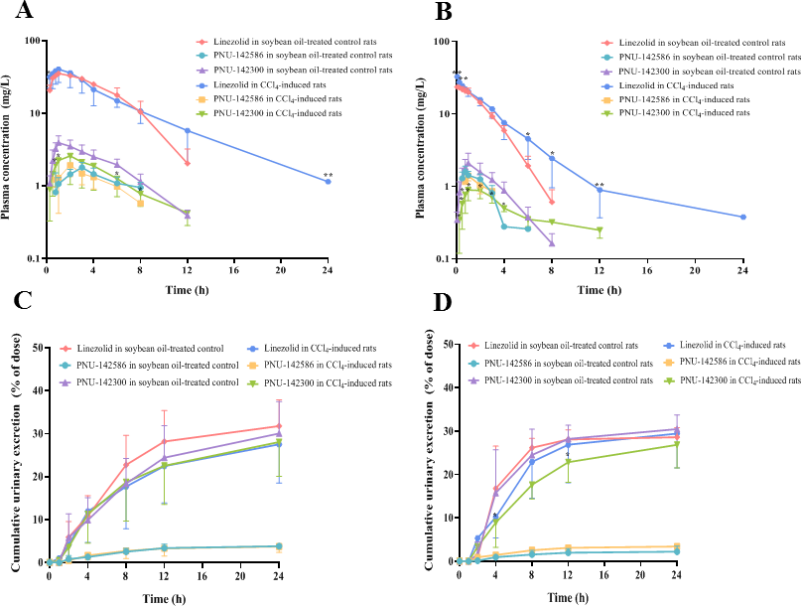
Mean plasma concentration-time curves and cumulative urinary excretion profiles of linezolid, PNU-142586, and PNU-142300 after i.g. and i.v. administration of linezolid to soybean oil-treated control and CCl_4_-induced rats. Mean plasma concentration-time curve of linezolid, PNU-142586, and PNU-142300 after i.g. administration of linezolid (63 mg/kg) (**A**), mean plasma concentration-time curve of linezolid, PNU-142586, and PNU-142300 after i.v. administration of linezolid (25 mg/kg) (**B**), cumulative urinary excretion profile of linezolid, PNU-142586, and PNU-142300 after i.g. administration of linezolid (63 mg/kg) (**C**), cumulative urinary excretion profile of linezolid, PNU-142586, and PNU-142300 after i.v. administration of linezolid (25 mg/kg) (**D**). Each point was presented as mean ± SD (*n* = 5 for each group). *P < 0.05, **P < 0.01 compared with the control group.

**Fig 4:**
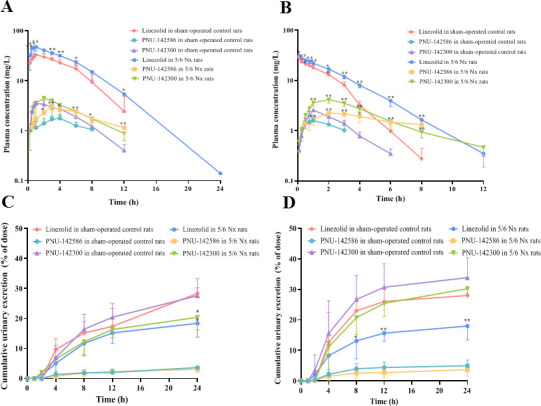
Mean plasma concentration-time curves and cumulative urinary excretion profiles of linezolid, PNU-142586, and PNU-142300 after i.g. and i.v. administration of linezolid to sham-operated control and 5/6 Nx rats. Mean plasma concentration-time curve of linezolid, PNU-142586, and PNU-142300 after i.g. administration of linezolid (63 mg/kg) (**A**), mean plasma concentration-time curve of linezolid, PNU-142586, and PNU-142300 after i.v. administration of linezolid (25 mg/kg) (**B**), cumulative urinary excretion profile of linezolid, PNU-142586, and PNU-142300 after i.g. administration of linezolid (63 mg/kg) (**C**), cumulative urinary excretion profile of linezolid, PNU-142586, and PNU-142300 after i.v. administration of linezolid (25 mg/kg) (**D**). Each point was presented as mean ± SD (*n* = 5 for each group). *P < 0.05, **P < 0.01 compared with control rats.

The mean plasma concentration-time curves and cumulative urinary excretion profiles of linezolid, PNU-142586, and PNU-142300 after i.g. (63 mg/kg) or i.v. (25 mg/kg) administration of linezolid in soybean oil-treated control, CCl_4_-induced, sham-operated control, and 5/6 Nx rats are shown in Fig. 3 and 4. The pharmacokinetic parameters are listed in Tables 2 and 3. After i.g. administration, linezolid was rapidly and completely absorbed. Linezolid was the main circulating component in plasma. The cumulative urinary excretion rates of linezolid, PNU-142586, and PNU-142300 were 30.0%, 3.5%, and 30.0%, respectively, in control rats.

**TABLE 2:** Pharmacokinetic parameters of linezolid, PNU-142586, and PNU-142300 after i.g. (63 mg/kg) or i.v. (25 mg/kg) administration of linezolid to control and CCl_4_-induced rats^*a*^

Route of administration	Pharmacokinetic parameters	Linezolid		PNU-142586		PNU-142300	
		Soybean oil-treated control rats	CCl_4_-induced rats	Soybean oil-treated control rats	CCl_4_-induced rats	Soybean oil-treated control rats	CCl_4_-induced rats
i.g.	AUC_0-∞_ (mg·h/L)	220.1 ± 27.6	269.2 ± 59.0^*^	14.4 ± 3.70	12.9 ± 0.96	23.8 ± 3.80	17.9 ± 7.90
	t_max_ (h)	1.0	1.0	2.80 ± 0.45	2.50 ± 1.00	1.20 ± 0.45	2.20 ± 1.10
	C_max_ (mg/L)	35.3 ± 1.10	40.4 ± 13.8	1.86 ± 0.21	2.00 ± 0.47	3.95 ± 0.94	2.62 ± 1.51
	t_1/2_ (h)	1.84 ± 0.27	3.50 ± 0.52^**^	4.31 ± 0.52	5.70 ± 1.16^*^	2.65 ± 0.28	3.81 ± 0.29^**^
	CL (L/h/kg)	0.29 ± 0.04	0.25 ± 0.07	—	—	—	—
	CL_r_ (L/h/kg)	0.02 ± 0.003	0.013 ± 0.003^*^	0.039 ± 0.01	0.041 ± 0.01	0.19 ± 0.11	0.22 ± 0.05
	CL_nr_ (L/h/kg)	0.27 ± 0.04	0.23 ± 0.06	—	—	—	—
	V_d_ (L/kg)	0.76 ± 0.03	1.23 ± 0.34^**^	—	—	—	—
	F (%)	115.8 ± 17.7	114.0 ± 29.3	—	—	—	—
i.v.	AUC_0-∞_ (mg·h/L)	69.3 ± 8.67	96.1 ± 21.4^*^	6.53 ± 1.44	5.64 ± 0.91	7.66 ± 2.29	6.14 ± 0.93
	t_max_ (h)	—	—	0.70 ± 0.11	1.20 ± 0.45	0.95 ± 0.11	1.15 ± 0.49
	C_max_ (mg/L)	—	—	1.69 ± 0.23	1.43 ± 0.20	2.15 ± 0.74	0.98 ± 0.23
	t_1/2_ (h)	1.21 ± 0.15	2.46 ± 0.32^**^	2.11 ± 0.42	2.97 ± 0.53^*^	1.57 ± 0.31	3.83 ± 1.21^**^
	CL (L/h/kg)	0.37 ± 0.04	0.27 ± 0.07^*^	—	—	—	—
	CL_r_ (L/h/kg)	0.02 ± 0.005	0.017 ± 0.009	0.043 ± 0.03	0.076 ± 0.0	0.22 ± 0.07	0.25 ± 0.11
	CL_nr_ (L/h/kg)	0.34 ± 0.038	0.26 ± 0.06	—	—	—	—
	V_d_ (L/kg)	0.63 ± 0.05	0.96 ± 0.25^*^	—	—	—	—

^*a*^ Data are expressed as mean ± SD (*n* = 5 for each group). “—”, Uncalculated null value; AUC_0-∞_, the area under the plasma concentration-time curve from time 0 to infinity; t_max_, time to maximum concentration; C_max_, maximum concentration; t_1/2_, elimination half-life; CL, total clearance; CL_r_, renal clearance; CL_nr_, non-renal clearance; V_d_, apparent volume of distribution; F, bioavailability. *P < 0.05, **P < 0.01 compared with the control group.

**TABLE 3:** Pharmacokinetic parameters of linezolid, PNU-142586, and PNU-142300 after i.g. (63 mg/kg) or i.v. (25 mg/kg) administration of linezolid to control and 5/6 Nx rats^*a*^

Route of administration	Pharmacokinetic parameters	Linezolid		PNU-142586		PNU-142300	
		Sham-operated control rats	5/6 Nx rats	Sham-operated control rats	5/6 Nx rats	Sham-operated control rats	5/6 Nx rats
i.g.	AUC_0-∞_ (mg·h/L)	210.3 ± 37.4	319.2 ± 34.5^**^	15.7 ± 2.89	29.5 ± 7.37^**^	23.9 ± 3.96	34.5 ± 10.5^**^
	t_max_ (h)	1.0	0.90 ± 0.14	3.60 ± 0.55	3.0	1.60 ± 0.55	2.20 ± 0.45^**^
	C_max_ (mg/L)	33.2 ± 2.10	49.3 ± 10.2^*^	1.92 ± 0.25	3.05 ± 0.52^**^	3.57 ± 0.37	4.51 ± 2.02^*^
	t_1/2_ (h)	2.05 ± 1.08	3.23 ± 0.72^*^	4.33 ± 0.32	4.98 ± 0.59^*^	2.61 ± 0.41	4.19 ± 0.82^**^
	CL (L/h/kg)	0.31 ± 0.05	0.20 ± 0.02^**^	—	—	—	—
	CL_r_ (L/h/kg)	0.02 ± 0.01	0.01 ± 0.003^*^	0.03 ± 0.01	0.02 ± 0.01*	0.15 ± 0.03	0.09 ± 0.02*
	CL_nr_ (L/h/kg)	0.29 ± 0.05	0.19 ± 0.02	—	—	—	—
	V_d_ (L/kg)	0.86 ± 0.32	0.92 ± 0.21	—	—	—	—
	F (%)	116.6 ± 20.1	93.3 ± 7.20	—	—	—	—
i.v.	AUC_0-∞_ (mg·h/L)	58.8 ± 2.26	90.6 ± 9.85^**^	6.31 ± 1.74	17.8 ± 4.89^**^	8.36 ± 1.23	23.4 ± 1.86^**^
	t_max_ (h)	—	—	0.85 ± 0.14	2.60 ± 0.89^*^	1.0	1.80 ± 0.45^*^
	C_max_ (mg/L)	—	—	1.67 ± 0.41	2.43 ± 0.16^*^	2.58 ± 0.20	4.17 ± 0.59^**^
	t_1/2_ (h)	1.16 ± 0.24	1.72 ± 0.29^*^	2.53 ± 0.15	3.88 ± 1.05^*^	1.62 ± 0.21	2.62 ± 0.88^*^
	CL (L/h/kg)	0.43 ± 0.02	0.28 ± 0.03^**^	—	—	—	—
	CL_r_ (L/h/kg)	0.03 ± 0.01	0.01 ± 0.003^**^	0.03 ± 0.01	0.02 ± 0.01^*^	0.21 ± 0.04	0.09 ± 0.02^**^
	CL_nr_ (L/h/kg)	0.40 ± 0.012	0.27 ± 0.03	—	—	—	—
	V_d_ (L/kg)	0.71 ± 0.14	0.69 ± 0.08	—	—	—	—

^*a*^ Data are expressed as mean ± SD (*n* = 5 for each group). “—”, Uncalculated null value; AUC_0-∞_, the area under the plasma concentration-time curve from time 0 to infinity; t_max_, time to maximum concentration; C_max_, maximum concentration; t_1/2_, elimination half-life; CL, total clearance; CL_r_, renal clearance; CL_nr_, non-renal clearance; V_d_, apparent volume of distribution; F, bioavailability. *P < 0.05, **P < 0.01 compared with the control group.

In the CCl_4_-induced rats, CL of linezolid was decreased by 13.8% and 27.0% in comparison to the soybean oil-treated control rats after i.g. and i.v. administration, respectively. This resulted in significantly higher AUC_0-∞_ values (1.22- and 1.39-fold) and t_1/2_ (1.90- and 2.03-fold) in CCl_4_-induced rats in comparison to the control rats ( *P* < 0.05) after i.g. and i.v. administration. The AUC_0-∞_ of PNU-142586 was decreased by 10.4% and 13.6%, while t_1/2_ was significantly prolonged by 1.32- and 1.41-fold compared to those in soybean oil-treated control rats after i.g. and i.v. administration of linezolid, respectively (*P* < 0.05). The AUC_0-∞_ of PNU-142300 was decreased by 24.8% and 19.8%, while t_1/2_ was significantly prolonged by 1.44- and 2.44-fold compared to those in soybean oil-treated control rats after i.g. and i.v. linezolid administration, respectively (*P* < 0.01).

In 5/6 Nx rats, the CL_r_ of linezolid was significantly decreased by 50.0% and 66.7% in comparison to the sham-operated control rats after i.g. and i.v. administration, respectively. CL was also significantly decreased (*P* < 0.01). This resulted in significantly higher AUC_0-∞_ (1.52- and 1.54-fold) and t_1/2_ (1.58- and 1.48-fold) values in 5/6 Nx rats in comparison to the control rats (*P* < 0.05) after i.g. and i.v. administration. AUC_0-∞_ of PNU-142586 was significantly increased by 1.88- and 2.82-fold, t_1/2_ was significantly prolonged by 1.15- and 1.53-fold, and CL_r_ was significantly decreased by 33.3% and 33.3% than those in sham-operated control rats after i.g. and i.v. administration of linezolid, respectively (*P* < 0.05). The AUC_0-∞_ of PNU-142300 was significantly increased by 1.44- and 2.80-fold, t_1/2_ was significantly prolonged by 1.61- and 1.62-fold, and CL_r_ was significantly decreased by 40.0% and 57.1% than those in sham-operated control rats after i.g. and i.v. linezolid administration, respectively (*P* < 0.01). In addition, the cumulative urinary excretion rates of linezolid, PNU-142586, and PNU-142300 were significantly decreased in 5/6 Nx rats (*P* < 0.05).

There were no significant changes in t_max_ and bioavailability of linezolid in the control, CCl_4_-induced, and 5/6 Nx rats following i.g. administration of linezolid (*P* > 0.01). Compared to the control rats, CL, CL_r_, and non-renal clearance (CL_nr_) of linezolid were lower in CCl₄-induced rats and 5/6 Nx rats, which may be attributed to reduced hepatic metabolism and renal excretion.

### Effects of impaired hepatic function or renal failure on the tissue distribution of linezolid, PNU-142586, and PNU-142300

**Fig 5:**
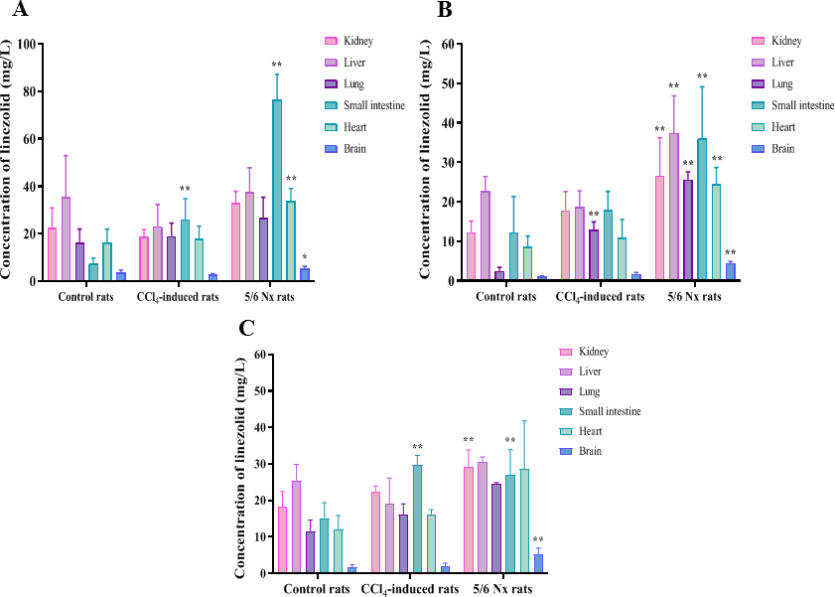
Tissue distribution of linezolid in control, CCl_4_-induced, and 5/6 Nx rats at 0.5 h (**A**), 1 h (**B**), and 4 h (**C**) after i.g. administration of linezolid (63 mg/kg). Each point was presented as mean ± SD (*n* = 4 for each group). *P < 0.05, **P < 0.01 compared with control rats.

**Fig 6:**
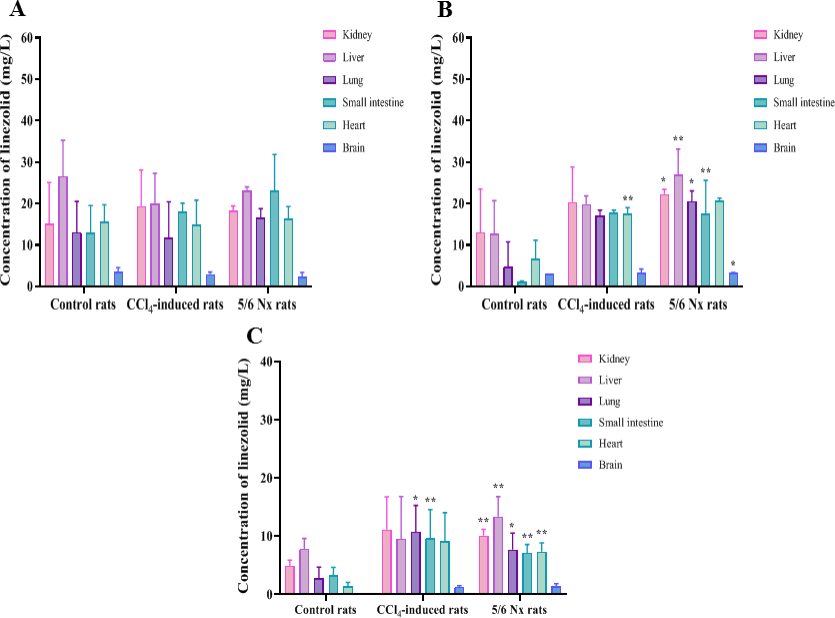
Tissue distribution of linezolid in control, CCl_4_-induced, and 5/6 Nx rats at 0.5 h (**A**), 1 h (**B**), and 4 h (**C**) after i.v. administration of linezolid (25 mg/kg). Each point was presented as mean ± SD (*n* = 4 for each group). *P < 0.05, **P < 0.01 compared with control rats.

The tissue distribution of linezolid after i.g. (63 mg/kg) or i.v. (25 mg/kg) administration of linezolid in the control, CCl_4_-induced, and 5/6 Nx rats are shown in Fig. 5 and 6. The tissue/plasma concentration ratios of linezolid and PNU-142300 are listed in Tables 4 and 5. After i.g. and i.v. administration, linezolid was widely distributed. In the control rats, concentrations of linezolid in the kidney and liver were higher than or comparable to those in the plasma, while the concentrations in the lung, small intestine, and heart were lower than in the plasma at all sampling time points. No PNU-142586 was detected in the brain, heart, lung, liver, kidney, or small intestine tissues. No PNU-142300 was detected in the kidney, while higher liver/plasma concentration ratios for PNU-142300 were observed.

**TABLE 4:** Tissue/plasma concentration ratios of linezolid and PNU-142300 after i.g. administration of linezolid (63 mg/kg) to control, CCl_4_-induced, and 5/6 Nx rats^*a*^

Time (h)	Linezolid			PNU-142300		
Kidney	Control rats	CCl_4_-induced rats	5/6 Nx rats	Control rats	CCl_4_-induced rats	5/6 Nx rats
0.5	1.05 ± 0.34	0.85 ± 0.16	1.07 ± 0.34	ND	2.32 ± 0.39	2.79 ± 0.61
1	0.42 ± 0.06	1.18 ± 0.22^**^	0.94 ± 0.18^**^	ND	3.83 ± 0.32	2.90 ± 1.51
4	0.75 ± 0.15	1.11 ± 0.17^*^	0.97 ± 0.25	ND	1.72 ± 0.25	3.09 ± 0.90
Liver						
0.5	1.64 ± 0.75	1.06 ± 0.44	1.25 ± 0.60	4.00 ± 1.55	ND	3.37 ± 1.14
1	0.78 ± 0.14	1.19 ± 0.18^*^	1.37 ± 0.41^*^	1.41 ± 1.04	ND	2.50 ± 1.08
4	1.06 ± 0.16	0.93 ± 0.31	1.00 ± 0.11	2.79 ± 1.08	ND	1.91 ± 0.34
Lung						
0.5	0.67 ± 0.13	0.68 ± 0.16	0.86 ± 0.38	ND	ND	ND
1	0.07 ± 0.03	0.66 ± 0.05^**^	0.94 ± 0.18^**^	ND	ND	ND
4	0.46 ± 0.15	0.60 ± 0.04	0.82 ± 0.09^**^	ND	ND	ND
Small intestine						
0.5	0.35 ± 0.21	0.93 ± 0.26^*^	2.48 ± 0.76^**^	ND	ND	ND
1	0.33 ± 0.22	0.90 ± 0.09^**^	1.28 ± 0.29^**^	ND	ND	ND
4	0.60 ± 0.18	1.11 ± 0.07^**^	0.90 ± 0.31	ND	ND	ND
Heart						
0.5	0.68 ± 0.06	0.64 ± 0.14	1.08 ± 0.24^*^	ND	ND	ND
1	0.24 ± 0.06	0.54 ± 0.18^*^	0.88 ± 0.12^**^	ND	ND	ND
4	0.47 ± 0.15	0.61 ± 0.03	0.96 ± 0.47	ND	ND	ND
Brain						
0.5	0.15 ± 0.02	0.10 ± 0.01^**^	0.17 ± 0.06	ND	ND	ND
1	0.03 ± 0.002	0.09 ± 0.02^*^	0.16 ± 0.03^**^	ND	ND	ND
4	0.06 ± 0.03	0.07 ± 0.04	0.18 ± 0.06^*^	ND	ND	ND

^*a*^ Data are expressed as mean ± SD (*n* = 4 for each group). *P < 0.05, **P < 0.01 compared with the control group. ND, less than the lower limits of quantification (0.25 mg/L).

**TABLE 5:** Tissue/plasma concentration ratios of linezolid and PNU-1423000 after i.v. administration of linezolid (25 mg/kg) to control, CCl_4_-induced, and 5/6 Nx rats^*a*^

Time (h)	Linezolid			PNU-142300		
Kidney	Control rats	CCl_4_-induced rats	5/6 Nx rats	Control rats	CCl_4_-induced rats	5/6 Nx rats
0.5	0.64 ± 0.36	1.28 ± 0.56	0.97 ± 0.12	ND	5.17 ± 3.53	2.51 ± 0.53
1	0.62 ± 0.52	1.08 ± 0.47	0.92 ± 0.18	ND	1.44 ± 1.40	3.86 ± 3.82
4	0.84 ± 0.14	1.14 ± 0.31	1.00 ± 0.23	ND	0.86 ± 0.30	2.61 ± 1.37
Liver						
0.5	1.22 ± 0.46	1.32 ± 0.36	1.24 ± 0.21	3.60 ± 1.67	ND	2.58 ± 0.67
1	0.60 ± 0.38	1.04 ± 0.15^*^	1.11 ± 0.34^*^	3.02 ± 1.87	ND	3.35 ± 1.85
4	1.30 ± 0.36	1.19 ± 0.85	1.39 ± 0.40	1.32 ± 0.04	ND	2.04 ± 0.96
Lung						
0.5	0.42 ± 0.25	0.55 ± 0.34	0.87 ± 0.03	ND	ND	ND
1	0.25 ± 0.18	0.66 ± 0.08^**^	0.91 ± 0.08^**^	ND	ND	ND
4	0.45 ± 0.46	0.85 ± 0.14	0.74 ± 0.20^*^	ND	ND	ND
Small intestine						
0.5	0.42 ± 0.21	0.66 ± 0.05	1.19 ± 0.31^**^	ND	ND	ND
1	0.21 ± 0.01	0.69 ± 0.05^**^	0.77 ± 0.34	ND	ND	ND
4	0.47 ± 0.42	0.71 ± 0.09	0.73 ± 0.07	ND	ND	ND
Heart						
0.5	0.50 ± 0.09	0.63 ± 0.05	0.85 ± 0.04^**^	ND	ND	ND
1	0.40 ± 0.07	0.67 ± 0.05^**^	0.91 ± 0.04^**^	ND	ND	ND
4	0.13 ± 0.10	0.67 ± 0.09^*^	0.75 ± 0.13^**^	ND	ND	ND
Brain						
0.5	0.11 ± 0.02	0.16 ± 0.12	0.12 ± 0.04	ND	ND	ND
1	0.12 ± 0.02	0.12 ± 0.03	0.15 ± 0.01	ND	ND	ND
4	ND	0.08 ± 0.01	0.13 ± 0.04	ND	ND	ND

^*a*^ Data are expressed as mean ± SD (*n* = 4 for each group). *P < 0.05, **P < 0.01 compared with the control group. ND, less than the lower limits of quantification (0.25 mg/L).

In the CCl_4_-induced rats, the tissue/plasma ratios of linezolid were higher than those in control rats after i.g. and i.v. administration of linezolid. However, no time-dependent increase in the tissue/plasma concentration ratios was observed within 4 hours after administration. And PNU-142300 was not detected in the liver. Only higher kidney/plasma concentration ratios for PNU-142300 were observed.

In 5/6 Nx rats, the tissue/plasma ratios of linezolid were also higher than those in control rats. After i.g. and i.v. administration, the renal linezolid concentrations in 5/6 Nx rats were approximately one-fold to two-fold higher than those in control rats. However, no time-dependent increase in the tissue/plasma concentration ratios was observed within 4 hours after administration. In addition, higher concentrations of PNU-142300 were detected in the kidney of 5/6 Nx rats than in control rats. The liver/plasma concentration ratios of PNU-142300 in 5/6 Nx rats were comparable with those in control rats.

### Incubation of linezolid with primary rat hepatocytes

**Fig 7:**
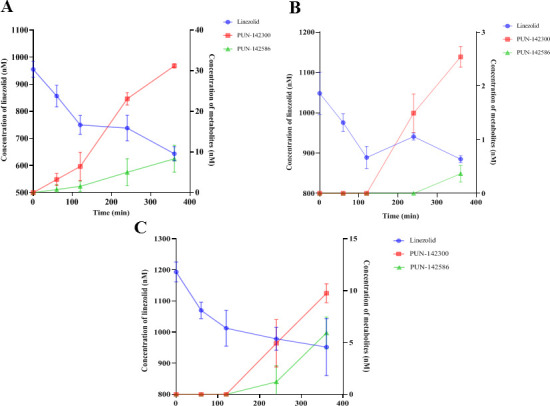
The concentration-time curves of linezolid, PNU-142586, and PNU-142300 after incubation of normal, CCl_4_-induced, and 5/6 Nx rat hepatocytes with linezolid (1 µM). The concentration-time curves of linezolid, PNU-142586, and PNU-142300 after incubation in normal hepatocytes (**A**), the concentration-time curves of linezolid, PNU-142586, and PNU-142300 after incubation in CCl_4_-induced rat hepatocytes (**B**), and the concentration-time curves of linezolid, PNU-142586, and PNU-142300 after incubation in 5/6 Nx rat hepatocytes (**C**). The results are presented as mean ± SD (*n* = 3). *P < 0.05, **P < 0.01 compared with normal group.

The effects of impaired hepatic function or renal failure on the hepatic metabolism of linezolid (1 µM) are shown in Fig. 7. No reductions in testosterone, 7-hydroxycoumarin, or linezolid levels were observed in the control, suggesting that the test substrate was stable during incubation. After substrate incubation with freshly isolated normal rat hepatocytes for 6 h, the remaining percentages of testosterone and 7-hydroxycoumarin were less than 2% and 5%, respectively. This suggests that the test system is suitable for this study.

Compared to the normal rat hepatocytes, the formation of PNU-142586 and PNU-142300 decreased by 95.6% and 91.9 %, respectively, after incubation for 6 h with the hepatocytes obtained from CCl_4_-induced rats, whereas the residue of linezolid increased by 25.3%. Compared to the normal rat hepatocytes, the formation of PNU-142586 and PNU-142300 decreased by 28.9% and 68.8%, respectively, after incubation with 5/6 Nx rat hepatocytes for 6 h, whereas the residue of linezolid increased by 18.4%. The t_1/2_ values of linezolid were 697.0, 1798.0, and 1247.3 min in normal, CCl_4_-induced, and 5/6 Nx rat hepatocytes, respectively. The CL_int, *in vitro*_ linezolid levels in normal, CCl_4_-induced, and 5/6 Nx rat hepatocytes were 4.65, 1.80, and 2.60 mL/min/kg, respectively. Linezolid metabolism in hepatocytes from rats with impaired hepatic function was significantly lower than that in normal rat hepatocytes (*P* < 0.01). Decreased activity of metabolic enzymes following hepatic impairment or renal failure affects linezolid metabolism.

### Uptake of linezolid by OATP1B1, OATP1B3, OATP2B1, NTCP, OAT1, and OAT3

**Fig 8:**
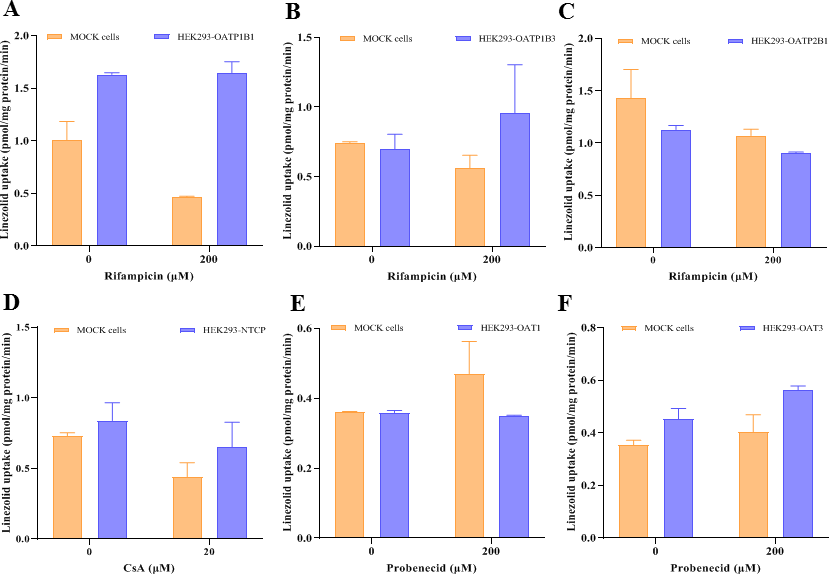
Uptake of linezolid in OATP1B1-, OATP1B3-, OATP2B1-, NTCP-, OAT1-, and OAT3-transfected HEK293 cells. The uptake of linezolid (5 µM) in mock and transporter-transfected HEK293 cells was terminated after incubation at 37°C for 10 min. Uptake of linezolid with and without rifampicin (200 µM) in OATP1B1-transfected HEK293 cells (**A**), uptake of linezolid with and without rifampicin (200 µM) in OATP1B3-transfected HEK293 cells (**B**), uptake of linezolid with and without erlotinib (10 µM) in OATP2B1-transfected HEK293 cells (**C**), uptake of linezolid with and without CsA (20 µM) in NTCP-transfected HEK293 cells (**D**), uptake of linezolid with and without probenecid (200 µM) in OAT1-transfected HEK293 cells (**E**), and uptake of linezolid with and without probenecid (200 µM) in OAT3-transfected HEK293 cells (**F**). Data represent mean ± SD of triplicate experiments.

Uptake of linezolid by OATP1B1, OATP1B3, OATP2B1, NTCP, OAT1, and OAT3 is shown in Fig. 8. The uptake of probe substrates in transporter-transfected cells was significantly greater than that in the corresponding control cells and was significantly reduced in the presence of positive control inhibitors (Fig. S1). This indicated the presence of functional transporters in the assay system.

The uptake of linezolid (5 µM) in OATP1B1-, OATP1B3-, OATP2B1-, NTCP-, OAT1-, and OAT3-transfected HEK293 cells was similar to that in the respective control cells. This indicates that linezolid is not a substrate for OATP1B1, OATP1B3, OATP2B1, NTCP, OAT1, or OAT3.

### Efflux transport of linezolid by MDR1 and MRP2

**Fig 9:**
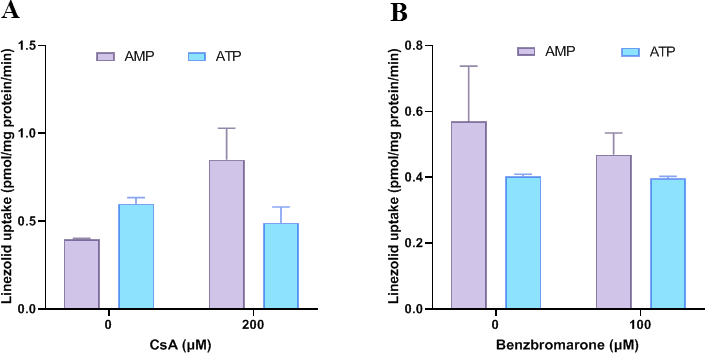
Uptake of linezolid in MDR1- and MRP2-containing membrane vesicles. The uptake of linezolid (5 µM) with or without ATP in MDR1- and MRP2-containing membrane vesicles was terminated after incubation at 37°C for 5 min. ATP-dependent uptake of linezolid with and without CsA (20 µM) in MDR1-containing membrane vesicles (**A**), ATP-dependent uptake of linezolid with and without benzbromarone (200 µM) in MRP2-containing membrane vesicles (**B**). Data represent mean ± SD of triplicate experiments.

Uptake of Linezolid by MDR1- and MRP2-containing membrane vesicles is shown in Fig. 9. The transport of probe substrates in ATP-containing membrane vesicles was significantly higher than that in the corresponding AMP-containing membrane vesicles, and the transport was significantly reduced in the presence of positive inhibitors (Fig. S2). This indicated the presence of functional transporters in the assay system. No significant ATP-dependent transport of linezolid (5 µM) was observed in MDR1- or MRP2-containing membrane vesicles, suggesting that linezolid is not a substrate for MDR1 or MRP2.

## DISCUSSION

Linezolid has complete oral bioavailability and good tissue penetration (27). It is used to treat infections in patients with liver or kidney disease. Recent studies have shown that liver or kidney injury is associated with increased linezolid exposure and thrombocytopenia following the standard dosage (30–33). However, there is no theoretical basis to support dose adjustment of linezolid in patients with hepatic impairment or renal failure. Understanding the effects and mechanisms of hepatic impairment and renal failure on the pharmacokinetics of linezolid and its two metabolites (PNU-142586 and PNU-142300) is critical for optimizing dosing strategies in such patients. In the present study, CCl_4_-induced and 5/6 Nx rat models were used to evaluate the effects of impaired hepatic function or renal failure on the pharmacokinetics of linezolid, PNU-142586, and PNU-142300 following i.g. or i.v. administration of linezolid. Isolated primary rat hepatocytes were used to evaluate the effects of impaired hepatic function or renal failure on linezolid metabolism *in vitro*. In addition, the roles of OATP1B1, OATP1B3, OATP2B1, NTCP, OAT1, OAT3, MDR1, and MRP2 in linezolid transport were evaluated. The results of this study will provide important information on the safe clinical use of linezolid.

Luque et al. conducted a 1:1 case-control study and found that patients with liver cirrhosis receiving standard doses of linezolid had higher median plasma concentrations than controls (20.6 mg/L vs 2.70 mg/L, *P* < 0.01) (38). Liao et al. found that the median trough concentration of linezolid in patients with severe hepatic impairment was significantly higher than those in patients with mild (20.7 mg/L vs 5.51 mg/L, *P* < 0.001) and moderate (20.7 mg/L vs 6.70 mg/L, *P* = 0.001) hepatic impairment (30). Furthermore, Tikiso et al. observed that the CL value of linezolid was significantly lower in a patient with acute-on-chronic liver failure compared to those without concomitant liver failure (1.54 L/h vs 6.26 L/h, *P* < 0.001) (39). Other studies have also indicated that hepatic impairment could increase plasma exposure to linezolid (31, 40, 41). The plasma exposure of linezolid and thrombocytopenia was increased in patients with renal impairment following standard linezolid dosing (32, 40, 42–44). In thrombocytopenic patients with moderate renal failure, the trough concentration of linezolid was 14.4–35.6 mg/L and the AUC_0-24_ was 513.1–994.6 mg·h/L (45), which was significantly higher than that of patients with normal renal function. Matsumoto et al. found that PNU-142586 and PNU-142300 accumulated in patients with renal failure (45). Some researchers advocate reducing the standard dose to 50% for patients with creatinine clearance (Cl_cr_) <60 mL/min/1.73 m^2^ (32, 46).

In this study, hepatic impairment and renal failure rat models were successfully established. There were no gender-related differences in the pharmacokinetics of linezolid in rats (12), so only male Sprague-Dawley rats were used in our study. Our results indicated that the influence of hepatic impairment or renal failure on the pharmacokinetics of linezolid and two metabolites did not differ between i.g. and i.v. administration in rats. Linezolid was the main circulating component in the plasma, PNU-142586, and PNU-142300 circulated at much lower concentrations and exhibited a delayed t_max_ compared to linezolid. PNU-142586 and PNU-142300 accounted for approximately 5.6%–8.5% and 9.2%–11.4% of the total plasma concentrations in control rats, respectively. The formation of PNU-142300 is the rate-limiting step in linezolid clearance. No significant changes in linezolid t_max_ or bioavailability were observed among control, CCl_4_-induced, or 5/6 Nx rats following i.g. administration of linezolid (*P* > 0.01), indicating that the absorption degree was not affected. CL, CL_r_, and CL_nr_ of linezolid were all decreased in CCl_4_-induced or 5/6 Nx rats, with a greater decrease observed in 5/6 Nx rats. AUC_0-∞,_ t_max_, C_max_, and CL_r_ of PNU-142586 and PNU-142300 showed no significant differences between CCl₄-induced and control rats. However, the AUC_0-∞_, C_max_, and t_1/2_ of PNU-142586 and PNU-142300 in 5/6 Nx rats were significantly increased (*P* < 0.05). Furthermore, no significant differences in the proportions of PNU-142586 and PNU-142300 were observed in the plasma of CCl₄-induced or 5/6 Nx rats.

In control rats, higher concentrations of linezolid were observed in the liver and kidney, while lower drug concentrations were observed in the lungs, heart, small intestine, and brain. This finding is consistent with previous reports (12, 47). No metabolites were detected in the brain, heart, lungs, or small intestine tissues of the control rats, and only a trace amount of PNU-142300 was found in the liver tissue. PNU-142586 was not detected in the tissues, possibly due to the insufficient sensitivity of the analytical method (the lower limit of quantification is 0.25 mg/L). Compared to the control rats, higher concentrations of PNU-142300 were detected in the kidney of CCl₄-induced and 5/6 Nx rats, while lower concentrations were observed in the liver of 5/6 Nx rats. No time-dependent increases in the kidney/plasma or liver/plasma concentration ratios were observed within 4 hours after i.g. and i.v. administration. Although the tissue/plasma concentration ratios for linezolid were higher in CCl_4_-induced or 5/6 Nx rats compared to control rats, linezolid did not accumulate in the brain, heart, lung, liver, kidney, or small intestinal tissues within 4 hours after i.g. and i.v. administration after hepatic impairment or renal failure. PNU-142300 did not accumulate in the liver or kidney tissue after hepatic impairment or renal failure. These results demonstrated that linezolid and its two major metabolites may have a lower risk of tissue accumulation in the context of hepatic or renal impairment.

Several studies have shown that linezolid in humans, rats, and dogs has similar pharmacokinetic characteristics, including 100% oral bioavailability, low protein binding, predominantly parent drug in plasma circulation, wide tissue distribution, slow oxidative metabolism to the same inactive metabolites, and significant urinary excretion (12, 14). The mean recovery of linezolid radioactivity in excreta was 93.8% in healthy volunteers, with 83.9% excreted in the urine and 9.9% in the feces (48). In humans, linezolid and PNU-142586 each accounted for more than 30% of dosed linezolid in the urine, and PNU-142300 accounted for approximately 9%. Feces predominantly contain metabolites, with only a small amount of linezolid parent drug. Linezolid metabolites in rat excreta are similar to those in humans (12). In rats, the mean recovery of linezolid radioactivity in excreta was over 95%, with 50%–60% of the dose detected in the urine and 20%–30% in feces. Rats favor the lactam pathway (lactone/lactam ratio 1:4), whereas humans favor the lactone pathway (lactone/lactam ratio 4.5:1) (48). At a given linezolid dose, higher metabolism via the lactam pathway may explain the lower AUC in rats than in humans. In our study, both linezolid and PNU-142300 were extensively excreted in the urine of control rats, accounting for approximately 30% of the administered dose. In CCl_4_-induced rats, the cumulative urinary excretion rates of linezolid, PNU-142586, and PNU-142300 showed no significant changes. By contrast, the cumulative urinary excretion rates of linezolid, PNU-142586, and PNU-142300 were significantly lower in 5/6 Nx rats (*P* < 0.05).

Liver metabolic enzyme activity decreases in CCl_4_-induced rats (37). Renal failure not only impairs renal elimination but also affects the non-renal disposition of drugs that are extensively metabolized by the liver (49–51). In this study, compared to the isolated normal rat hepatocytes, the *in vitro* hepatic clearance of linezolid in CCl_4_-induced or 5/6 Nx rat hepatocytes was decreased by 61.3% and 44.1%, respectively. The t_1/2_ values of linezolid in CCl_4_-induced or 5/6 Nx rat hepatocytes were 2.58- and 1.79-fold higher than that in normal rat hepatocytes. Hepatic metabolism was decreased, while renal excretion remained unchanged in CCl_4_-induced rats. Therefore, the AUC_0-∞_ values of linezolid in hepatic impairment rats were significantly higher than those in control rats, while the AUC_0-∞_ values of PNU-142586 and PNU-142300 were lower. The CL_r_/GFR ratios in healthy volunteers and rats were 0.4 and 0.08, respectively, suggesting that linezolid elimination may involve renal tubular reabsorption (12, 52). Allegra et al. found that *ABCB1* C3435T polymorphism had a significant effect on the pharmacokinetics of linezolid in 27 critically ill patients, speculating that linezolid might be a substrate of MDR1. However, Gandelman et al. argued that linezolid is not an MDR1 substrate, based on unpublished data from the linezolid manufacturer Pfizer (53). Cheli et al. found no association between linezolid exposure and *ABCB1* polymorphisms (54). The calculated basolateral-to-apical to apical-to-basolateral efflux ratio of linezolid was 1:1 in human MDR1-transfected Madin-Darby Canine Kidney cells (53). In addition, several studies have reported that linezolid is not a substrate for OCT1, OCT2, OCT3, MATE1, or MATE2-K (19, 20, 47). Our results indicate that linezolid is not a substrate for OATP1B1, OATP1B3, OATP2B1, NTCP, OAT1, OAT3, MDR1, or MRP2. Linezolid is neutral throughout the physiological pH range and may be taken up into enterocytes, hepatocytes, and renal epithelial cells via passive diffusion. A strong statistically significant correlation (r = 0.933, *P* < 0.01) was observed between linezolid clearance and creatinine clearance (CL_cr_) in humans (45). We speculated that renal clearance of linezolid was primarily through glomerular filtration, and renal tubular reabsorption was a minor route. Although the hepatic metabolism was reduced, the effect of reduced glomerular filtration was more significant in 5/6 Nx rats. AUC_0-∞_ values of linezolid, PNU-142586, and PNU-142300 in renal failure rats were all significantly higher than those in the control rats.

The mechanism underlying the development of linezolid-induced thrombocytopenia has not been fully clarified. It is reported that linezolid-induced thrombocytopenia is not caused by nonimmune-mediated bone marrow suppression (55). And PNU-142586 and PNU-142300 have cytotoxic effects on platelet precursor cell lines (megakaryocytic cells) (56). Linezolid-induced thrombocytopenia is associated with delayed elimination of PNU-142586 and PNU-142300 (56). PNU-142300 is a substrate for OAT3. In addition to reduced glomerular filtration, renal failure decreases OAT3 protein expression and activity, leading to increased plasma exposure to PNU-142586 and PNU-142300. Therefore, patients with renal failure may have a higher risk of thrombocytopenia than patients with hepatic impairment. A lower dosage of linezolid is necessary for patients with hepatic impairment or renal failure. In addition, careful monitoring of the concentrations of linezolid and these two metabolites is essential.

However, our study had some limitations that should be considered. First, although no significant change in the bioavailability of linezolid was observed after i.g. administration in control, CCl_4_-induced, and 5/6 Nx rats, it remains unclear whether hepatic impairment or renal failure would affect the absorption process of linezolid. Second, the feces and bile of rats were not collected during our study. The effect of hepatic impairment and renal failure on non-renal clearance could not be directly quantified. Third, the roles of transporters in the disposition of PNU-142586 and PNU-142300 were not analyzed. Therefore, future studies are needed to be conducted.

In this study, we found that linezolid and its two major metabolites may have a lower risk of tissue accumulation in the context of hepatic or renal impairment, despite altered pharmacokinetics. Renal clearance of linezolid was primarily eliminated by glomerular filtration, while tubular reabsorption via passive diffusion was a minor route. Hepatic metabolism was significantly reduced in rats with hepatic impairment, leading to increased plasma linezolid exposure and decreased metabolite levels. Renal failure significantly increased the plasma exposure of linezolid, PNU-142586, and PNU-142300 due to both decreased hepatic metabolism and reduced glomerular filtration. Considering these findings, dosage adjustment for linezolid in patients with hepatic impairment or renal failure is necessary.

## Data Availability

The raw and processed data required to reproduce the above findings are available to download from 10.6084/m9.figshare.28648799.

## SUPPLEMENTAL MATERIAL

Fig. S1 aac.01892-24-s0001.tif Uptake of specific substrates in OATP1B1-, OATP1B3-, OATP2B1-, NTCP-, OAT1-, and OAT3-transfected HEK293 cells.

Fig. S2 aac.01892-24-s0002.tif Uptake of specific substrates in MDR1- and MRP2-containing membrane vesicles.

Graphical abstract aac.01892-24-s0003.tif Visual diagram of study findings.

Supplemental legends aac.01892-24-s0004.docx Legends for Fig. S1 and S2.

ASM does not own the copyrights to Supplemental Material that may be linked to, or accessed through, an article. The authors have granted ASM a non-exclusive, world-wide license to publish the Supplemental Material files. Please contact the corresponding author directly for reuse.
