# Supplementary figures and images for: Impact of hepatic impairment and renal failure on the pharmacokinetics of linezolid and its metabolites: contribution of hepatic metabolism and renal excretion
Source: Antimicrob Agents Chemother. 2025 Apr 14;69(5):e01892-24. doi: 10.1128/aac.01892-24 (PMC12057336; doi:10.1128/aac.01892-24)

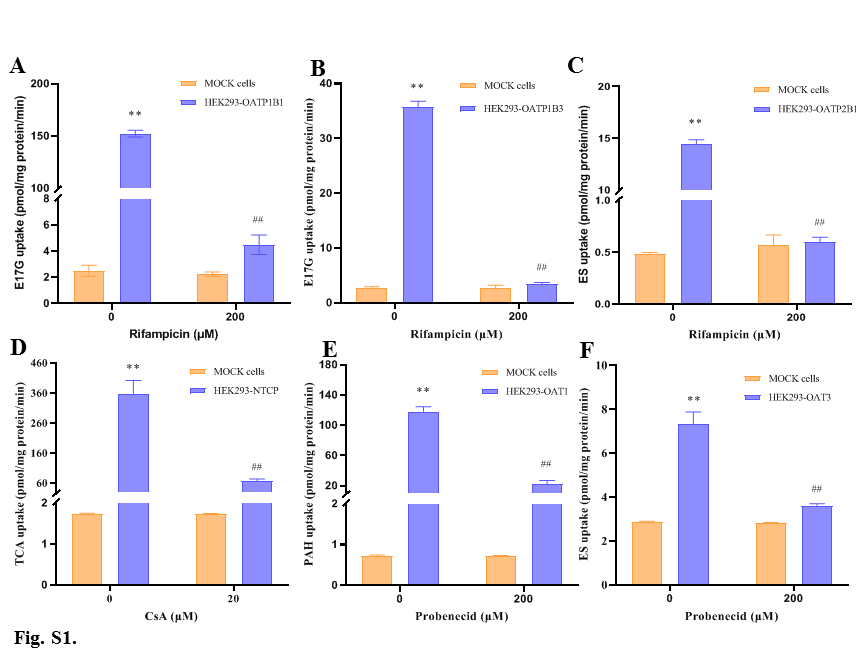

Supplement: Fig. S1 — Uptake of specific substrates in OATP1B1-, OATP1B3-, OATP2B1-, NTCP-, OAT1-, and OAT3-transfected HEK293 cells. [file aac.01892-24-s0001.tif]

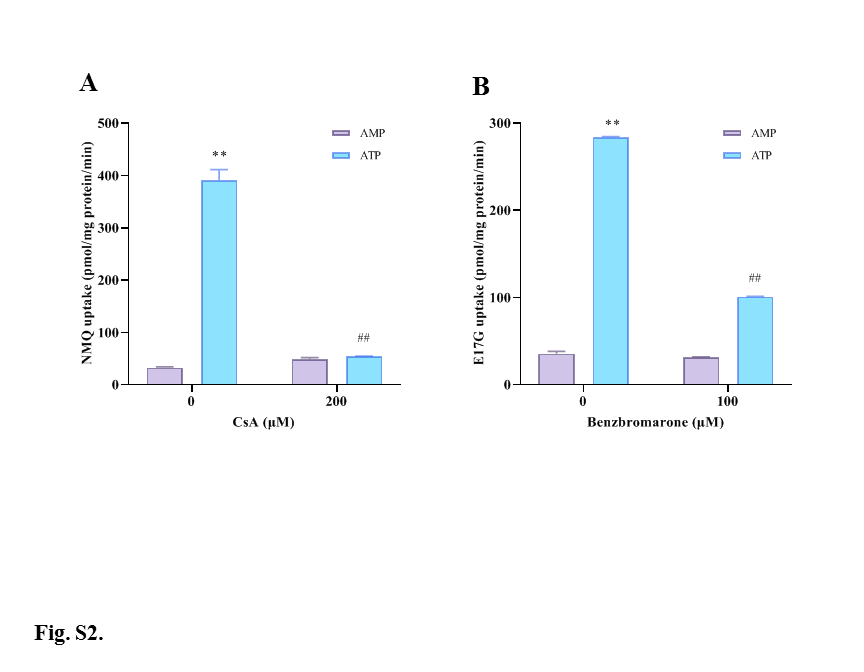

Supplement: Fig. S2 — Uptake of specific substrates in MDR1- and MRP2-containing membrane vesicles. [file aac.01892-24-s0002.tif]

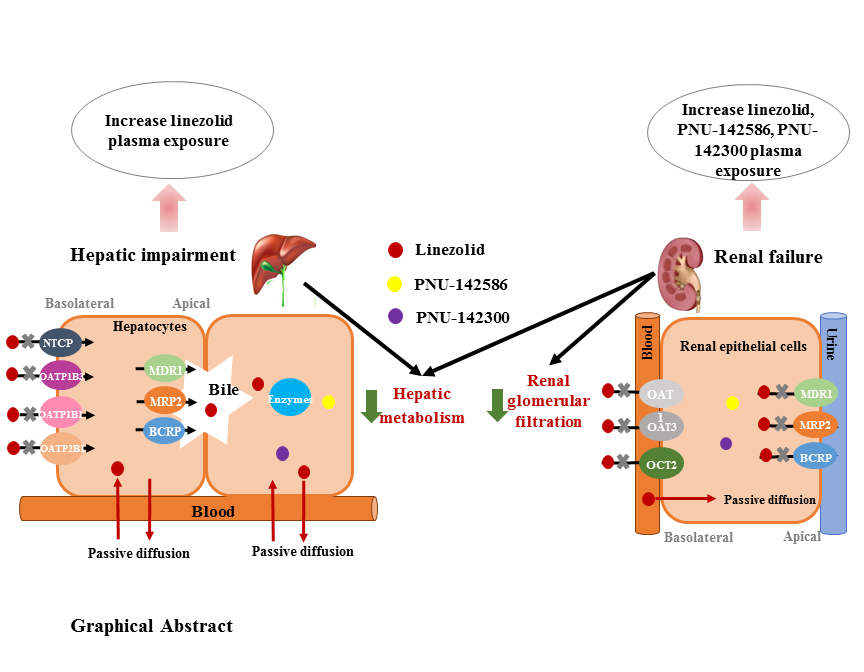

Supplement: Graphical abstract — Visual diagram of study findings. [file aac.01892-24-s0003.tif]
