# Supplementary material for: Impact of hepatic impairment and renal failure on the pharmacokinetics of linezolid and its metabolites: contribution of hepatic metabolism and renal excretion
Source: Antimicrob Agents Chemother. 2025 Apr 14;69(5):e01892-24. doi: 10.1128/aac.01892-24 (PMC12057336; doi:10.1128/aac.01892-24)
Supplement: Supplemental legends — Legends for Fig. S1 and S2. [file aac.01892-24-s0004.docx]

**Supplemental Material Legends**

**FIG S1** Uptake of specific substrates in OATP1B1-, OATP1B3-, OATP2B1-, NTCP-, OAT1- and OAT3-transfected HEK293 cells. The uptake of specific substrates in mock and transporter- transfected HEK293 cells was terminated after incubation at 37^◦^C for 10 min. Uptake of E17G (5 μM) with and without rifampicin (200 μM) in OATP1B1-transfected HEK293 cells (A), uptake of E17G (5 μM) with and without rifampicin (200 μM) in OATP1B3-transfected HEK293 cells (B), uptake of ES (2 μM) with and without erlotinib (10 μM), in OATP2B1-transfected HEK293 cells (C), uptake of TCA (10 μM) with and without CsA (20 μM) in NTCP-transfected HEK293 cells(D), uptake of PAH (20 μM) with and without probenecid (200 μM) in OAT1-transfected HEK293 cells (E), up-take of ES (5 μM) with and without probenecid (200 μM) in OAT3-transfected HEK293 cells (F). Data represents mean ± SD of triplicate experiments.

**FIG S2** Uptake of specific substrates in MDR1- and MRP2-containing membrane vesicles. The uptake of specific substrates with or without ATP in MDR1- and MRP2-containing membrane vesicles was terminated after incubation at 37^◦^C for 5 min. ATP-dependent uptake of NMQ (5 μM) with and without CsA (20 µM) in MDR1-containing membrane vesicles (A), ATP-dependent uptake of E17G (5 μM) with and without benzbromarone (200 µM) in MRP2-containing membrane vesicles (B). Data represents mean ± SD of triplicate experiments.
